# Sight and Perspective

**DOI:** 10.4269/ajtmh.18-0265

**Published:** 2018-10

**Authors:** Varsha Mona Puri

**Affiliations:** Children’s Hospital Los Angeles, Los Angeles, CA

The complaint that brings a patient in often masks something more serious. That was the case with Martha. Martha is a beautiful Ghanian woman. She bears the leather lifelines of 60 years of relentless farming, although they somehow decorate her face nicely. Her dress bestows the essence of Ghanian wear: a headwrap is neatly packaged on top of her head with an ornate, brightly patterned blouse and full-length skirt to match. Underneath this demeanor shines the spirit of a survivor: you see it in her eyes.

Today, she comes in complaining of falling twice in the last week. It is hard to imagine a woman with a lifetime practice of balancing her merchandise on her head and walking two miles that way to simply trip. In fact, that is hardly her case. She describes herself as having “weak knees.” But when you look closer, you see a cloudy haze covering those expressive eyes. Like many her age in Central Ghana, she is beginning to have cataracts. On asking, we find out that she can barely see me sitting right in front of her. For the first time in her life, she learns that she is hypertensive with her blood pressure at 160/100. Arthritis, cataracts, and hypertension are extremely common problems that plague our elderly, farming population in Ghana. There was no brilliance necessary in realizing that arthritis from 70 years of wear and tear on her knee joint along with her developing cataracts leaves her at risk for one mighty fall and a hip fracture to follow suit.

Hip fractures are reputedly tough for the elderly in the United States. However, we still rely on hip replacement surgery, well-trained anesthesiologists to handle age-related complications, and months of physiotherapy for rehab. If Martha has a fracture, coupled with her uncontrolled hypertension, her options are limited. Even if she is able to get surgery, she will no longer have an income and become reliant on someone to care for her, hence losing a second income.

This foresight has the head nurse, MaVic, and myself worried for her. We prod her to go to Agogo Hospital’s eye clinic. After all, she has free health insurance from the government (provided for the elderly). Her eyelids sag under the weight of her problems. She cannot afford to miss a day of work, nor can she afford the fare to Agogo Hospital ($0.75). The more we tell her to go, the heavier her eyes get. She looks away; shortly after, she checks out of the conversation.

We play out all the potentially dangerous prospects that lay in her immediate future. “What will you do when you can’t see?” We ask.

“Then I’ll just die,” she says.

That was not the direction I was hoping the conversation to go. Martha’s eyes are calling for help. But how much hope can you ask a person who has spent a lifetime fighting to put food in her mouth, who has spent a lifetime farming and selling vegetables at the expense of her body?

We find out that she has a daughter who lives nearby. She too will have to take a day off work; she too does not have fare for transportation, but she can take her. Now her eyes dance with uncertainty and a tinge of despair. In this aspect, Martha was more of a guilty mother than anything else: an older mother who strives to be independent of relying on her children. She really does not want to ask for help. Personally, I *hate* asking for help. So, perhaps only on this level, I understand her angst. However, Martha, MaVic, and I talk through it. Martha agrees to ask her.

Certain things in life cross all boundaries; they hit at the core in the most fundamental of human ways. Despair is universal; it is a feeling we all recognize. It is an uncontrollable situation: loss of control. My heart sunk under the weight of her increasingly cloudy eyes. I do not understand why she exists in her world under these circumstances and I under my “American” circumstance. Even under these circumstances, she came bearing vegetables for the clinic. “Why is it the people with the least amount always want to offer whatever they have and those who have more hold on tighter?” asked MaVic.

There is so much I do not understand, but this I do: life is much greater than myself and the visible world around me. I am grateful, if only for a brief period in time, to bear witness to that.

